# SETD7 is a prognosis predicting factor of breast cancer and regulates redox homeostasis

**DOI:** 10.18632/oncotarget.21583

**Published:** 2017-10-06

**Authors:** Run Huang, Xiaolin Li, Yang Yu, Lisi Ma, Sixuan Liu, Xiangyun Zong, Qi Zheng

**Affiliations:** ^1^ Department of Breast Surgery, Shanghai Jiao Tong University Affiliated Shanghai Sixth People’s Hospital, Shanghai 200233, China; ^2^ Department of Breast Surgery, Zhejiang Cancer Hospital, Hangzhou 310022, China; ^3^ Department of General Surgery, Shanghai Jiao Tong University Affiliated Shanghai Sixth People’s Hospital, Shanghai 200233, China

**Keywords:** SETD7, breast cancer, redox homeostasis, reactive oxygen species, oxidative stress

## Abstract

SETD7 is a methyltransferase that specifically catalyzes the monomethylation of lysine 4 on histone H3. A variety of studies has revealed the role of SETD7 in posttranslational modifications of non-histone proteins. However, the prognostic value of SETD7 on breast cancer and the ability of SETD7 of regulating intrinsic redox homeostasis has never been investigated. In this study, using The Cancer Genome Atlas (TCGA) database, we revealed that SETD7 was a potential prognostic marker of breast cancer. Median survival time of patients with low SETD7 expression (18.1 years) was twice than that of SETD7 low-expressed patients (9.5 years). We demonstrated that SETD7 promoted tumor cell proliferation and prevented cell apoptosis and that SETD7 delicately maintained the redox homeostasis through regulating the levels of GSH/GSSG and ROS. Further studies indicated that SETD7 was a positive activator of KEAP1-NRF2 pathway. Using dual luciferase assay, we revealed the role of SETD7 as a transcriptional activator of antioxidant enzymes. Downregulation of SETD7 in MCF7 and MDA-MB-231 cells impaired the expression of antioxidant enzymes and induces imbalance of redox status. Together, we proposed SETD7 as a prognostic marker of breast cancer and a novel antioxidant promoter under oxidative stress in breast cancer.

## INTRODUCTION

SETD7 (also known as SET7, SET9 and SET7/9), a SET domain containing lysine methyltransferase 7, specifically monomethylates lysine 4 at histone 3 (H3K4me1) and activates its involved genes expression [1]. Additionally, SETD7 has also been discovered to monomethylate a series of non-histone proteins, such as p53 [2], DNA cytosine methyltransferase 1 (DNMT1) [3], E2 promoter-binding factor 1 (E2F1) [4, 5], hypoxia-inducible factor 1α (HIF1α) [6, 7], YAP [8] and NF-κb [9]. These methylations lead to various and even contradictory results. For instance, Liu et al. revealed that glycolysis was inhibited due to the SETD7-induced methylation of HIF1α [6], while another study reported the opposite result [7]. Despite the regulating role of SETD7 on non-histone proteins, the function of SETD7 regulating redox state has never been intensively investigated in breast cancer.

Cellular redox homeostasis, which refers to the balance of reduction–oxidation balance, is of vital significance for tumor cells. Delicate mechanisms of regulating redox homeostasis lead to a well-balanced redox status and they synergize to promote tumor initiation, proliferation and migration under the circumstances of moderate reactive oxygen species (ROS) levels [10, 11]. ROS, the byproducts of oxygen metabolism, play vital roles in intracellular redox homeostasis, cellular signaling, immunity and aging [12]. However, most studies hold the opinion that high ROS levels are detrimental to cancer cells, because excessive ROS can induce DNA damage and apoptosis [13–16].

As ROS levels increase, cellular oxidative stress enhances, which gives rise a survival challenge to cancer cells [14]. Nevertheless, complex antioxidant pathways will be stimulated to increase expression of ROS scavengers, thus preventing ROS-induced cell death [10]. Particularly, the transcriptional factor nuclear factor E2-related factor 2 (NRF2), controls the expression of a series of antioxidant enzymes through binding to the antioxidant response element (ARE) in their promoter regions [17]. In non-stressed cells, the constitutively active Kelch-like ECH-associated protein 1 (KEAP1) promotes the ubiquitination and degradation of NRF2 in a cullin-3-dependent pathway [18]. Upon oxidative stress, KEAP1 is oxidized and NRF2 is accumulated. Then NRF2 translocates into the nucleus and trigger the antioxidant response [18].

Here in this study, we analyzed the expression data and clinical data downloaded from The Cancer Genome Atlas (TCGA). Using univariate and multivariate survival analysis, we determined SETD7 to be a possible prognostic marker of breast cancer. Moreover, we explored the correlation between SETD7 expression and KEAP1-NRF2 pathway and its downstream target genes, including malic enzyme 1 (ME1), thioredoxin reductase 1 (TXNRD1), and the catalytic subunit (GCLC) and modifier subunit (GCLM) of glutamate-cysteine ligase (GCL). Combined with the result of luciferase reporter assay, we concluded that SETD7 was a potential promoter of the antioxidant pathway counterbalancing the cytotoxic effect of oxidative stress, thereby inducing the poor prognosis of breast cancer.

## RESULTS

### SETD7 is a potential prognostic marker of breast cancer

In order to assess the function of SETD7 in breast cancer patients, we firstly analyzed the SETD7 expression data obtained from TCGA database. Breast cancer patients (n=1086) were divided into two cohorts according to the median of SETD7 expression. The median follow-up of survivors was 73.6 months (interquartile range: 37.6–140.2 months), with 149 deaths. To investigate the clinical relevance of SETD7 in breast cancer patients, we compared the distributions of differential clinicopathological characteristics based on SETD7 expression (Table 1). There were no differences on SETD7 expression came from age and menopause, nor the T, N and M of TNM stage. However, SETD7 expression seemed to be more highly expressed in White people than Asian and Black or African American persons. Particularly, there exists a trend that, in SETD7 highly-expressed patients, estrogen receptor and progesterone receptor expression were significantly higher than those patients with SETD7 low expression, while HER2 expression was lower when SETD7 was highly expressed.

**Table 1:** The relationship between SETD7 and clinicopathological characteristics of breast cancer patients in TCGA database

Factors	All	SETD7 low	SETD high	*P*	Missing data
Age, yr				0.1775	0
35-70	826 (76.1)	422 (77.7)	404 (74.4)		
≤35	35 (3.2)	20 (3.7)	15 (2.8)		
≥70	225 (20.7)	101 (18.6)	124 (22.8)		
Menopause				0.6663	121
Pre	265 (27.5)	128 (26.8)	137 (28.1)		
Post	700 (72.5)	349 (73.2)	351 (71.9)		
Race				<0.0001	96
White	748 (75.5)	349 (67.0)	399 (85.0)		
Asian	61 (6.2)	33 (6.3)	28 (6.0)		
Black or African American	181 (18.3)	139 (26.7)	42 (9.0)		
T stage				0.1714	2
1	276 (25.5)	131 (24.2)	145 (26.8)		
2	632 (58.3)	323 (59.6)	309 (57.0)		
3	137 (12..6)	74 (13.6)	63 (11.6)		
4	39 (3.6)	14 (2.6)	25 (4.6)		
N stage				0.2483	20
0	510 (47.8)	259 (48.5)	251 (47.2)		
1	360 (33.8)	174 (32.6)	186 (35.0)		
2	119 (11.2)	55 (10.3)	64 (12.0)		
3	77 (7.2)	46 (8.6)	31 (5.8)		
M stage				0.3113	161
0	903 (97.6)	435 (97.1)	468 (98.1)		
1	22 (2.4)	9 (2.9)	13 (1.9)		
ER				<0.0001	49
Negative	235 (22.7)	158 (30.6)	77 (14.8)		
Positive	802 (77.3)	359 (69.4)	443 (85.2)		
PR				<0.0001	52
Negative	341 (33.0)	207 (40.2)	134 (25.8)		
Positive	693 (67.0)	308 (59.8)	385 (74.2)		
HER2				0.0004	139
Negative	760 (80.3)	395 (85.0)	365 (75.7)		
Positive	187 (19.7)	70 (15.0)	117 (24.3)		

Values are expressed as n (%). ER, estrogen receptor; PR, progesterone receptor; HER2, human epidermal growth factor receptor-2. Missing data of all variates was no more than 16%. Chi square test was employed to analyze these data.

**Figure 1:**
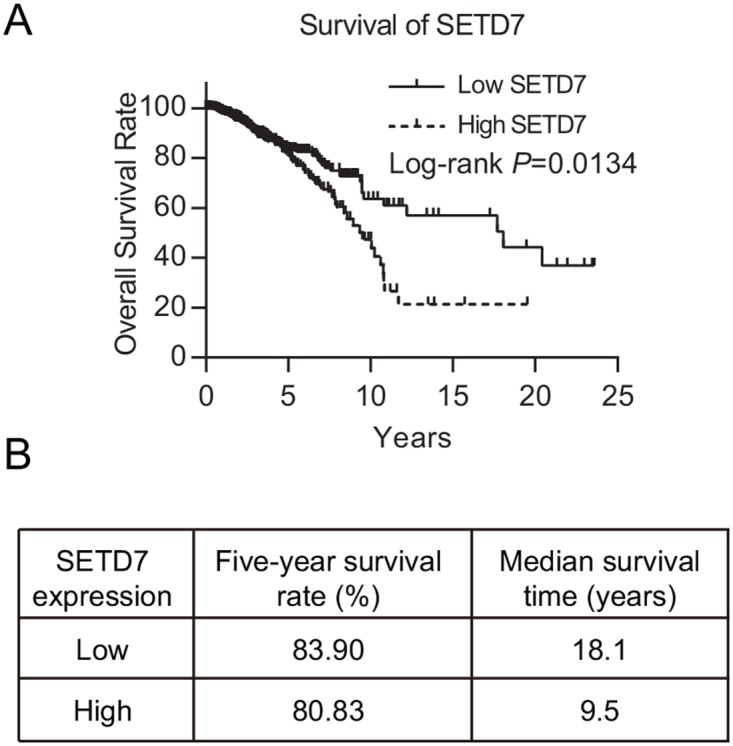
SETD7 predicts the prognosis of breast cancer **(A)** Kaplan-Meier survival plot of 1086 breast cancer patients with high and low SETD7 expression in TCGA database. **(B)** Five-year survival and median survival time were illustrated.

We used univariate and multivariate analysis to analyze the prognostic factors of overall survival (OS) for patients. In univariate analysis, we discovered that SETD7 expression, age, menopause, surgical margin status, and T and N factor of TNM stage were significantly associated with poorer OS (Table 2 and Figure 1). Higher expression of SETD7 predicted a much worse overall survival (Figure 1A). Although little difference of five-year survival rate was discovered between two cohorts, yet the median survival time of cohort with lower SETD7 expression was nearly twice longer than that of SETD7 higher expression cohort (Figure 1B). Significant indicators then entered the multivariate analysis to build a COX proportional hazard model and we found age, menopause and N factor of TNM stage to be independent prognostic factors of poor OS. Although SETD7 was not found to be an independent prognostic factor for breast cancer, these results revealed the prognostic significance of SETD7 for breast cancer patients, especially for their long-term survival.

**Table 2:** The relationship between clinicopathological characteristics and survival of breast cancer patients in TCGA database

	Univariate analysis		Multivariate analysis	
Factors	P-value	HR (95% CI)	P-value	HR (95% CI)
Age (35-70/≤35/≥70), yr	<0.0001	1.61 (1.35-1.92)	<0.0001	1.90 (1.10-3.29)
Menopause (pre/post)	0.0005	2.39 (1.43-3.98)	0.0221	2.01 (1.05-3.83)
Race (White/Asian/African American)	0.5334	-	-	-
Surgical margin (negative/positive)	0.0051	2.01 (1.22-3.32)	-	-
T stage (1/2/3/4)	<0.0001	1.44 (1.18-1.76)	-	-
N stage (0/1/2/3)	<0.0001	1.63 (1.38-1.94)	<0.0001	1.84 (1.51-2.24)
M stage (0/1)	0.5065	1.36 (0.55-3.33)		
ER (negative/positive)	0.0906	0.73 (0.51-1.05)	-	-
PR (negative/positive)	0.0906	0.75 (0.53-1.05)	-	-
HER2 (negative/positive)	0.5713	1.15 (0.71-1.88)	-	-
SETD7 (high/low)	0.0134	1.51 (1.09-2.09)	0.2848	1.24(0.84-1.84)

ER, estrogen receptor; PR, progesterone receptor; HER2, human epidermal growth factor receptor-2. Subgroups in the first place of each factor was referred to the reference group.

### SETD7 positively correlates with NRF2 target genes

**Figure 2:**
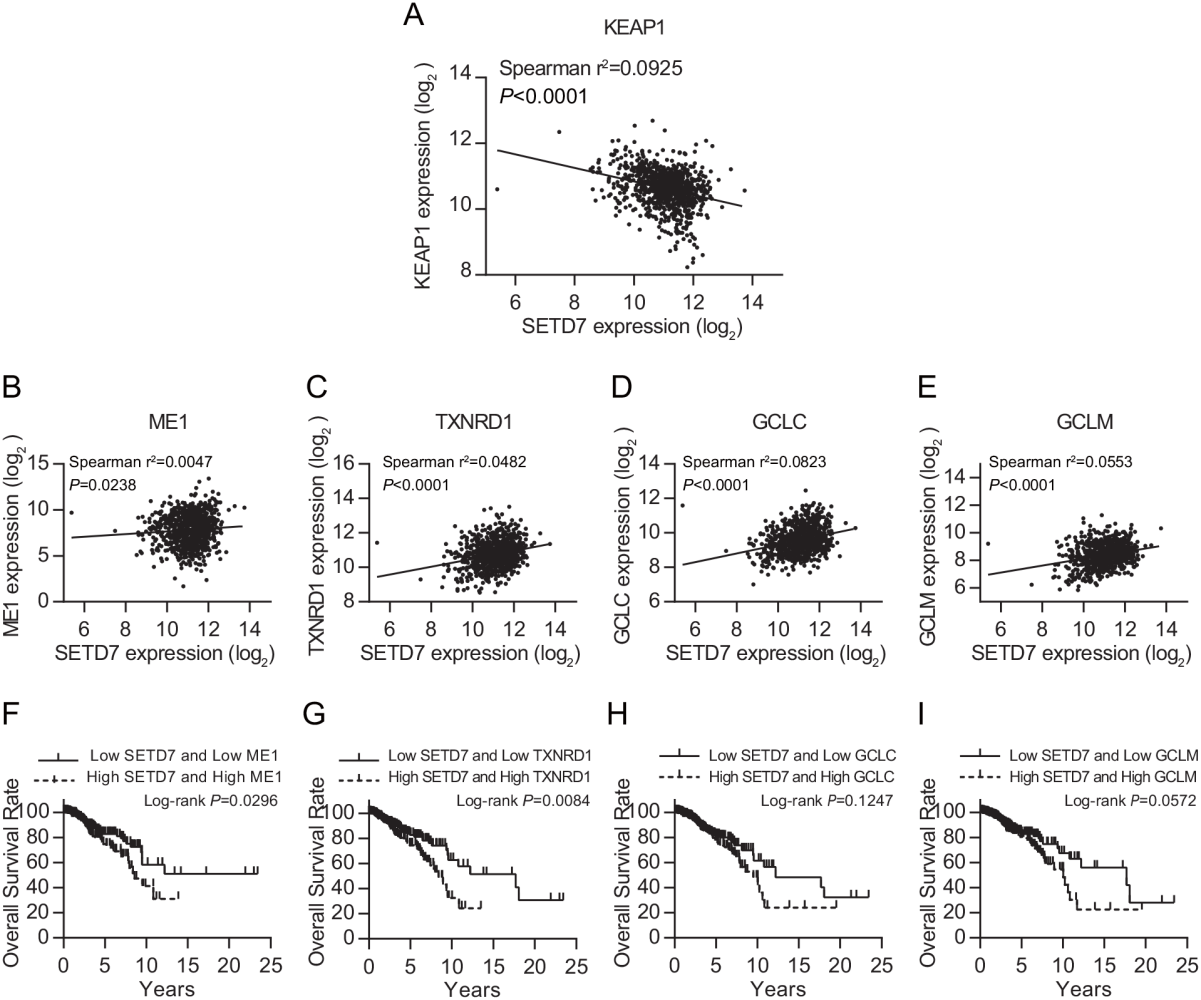
SETD7 is tightly correlated with KEAP1-NRF2 pathway **(A)** Negative correlation between SETD7 and KEAP1. **(B-E)** Positive correlation between SETD7 and NRF2 target genes. (**F-I)** SETD7 predicted breast cancer survival together with NRF2 target genes.

To investigate the molecular mechanism of SETD7 inducing poor outcome of breast cancer patients, we explored the expression data of breast cancer in TCGA database. Using Spearman correlation test, we found that SETD7 expression was potently and negatively correlated with the expression of the negative regulator of NRF2, KEAP1 (Figure 2A). Moreover, we analyzed the expression data of several NRF2 target genes (ME1, TXNRD1, GCLC and GCLM), which are important antioxidants, catalyzing the reduction and detoxification of cytotoxic ROS. All of the four NRF2 target genes were positively correlated with the expression of SETD7 (Figure 2B-2E). This might reveal the regulating effect of SETD7 on NRF2 target genes expression. Additionally, to evaluate the interaction effect between SETD7 and NRF2 target genes on breast cancer survival, we correlated their expression levels with patient outcome. Data analysis revealed that lower SETD7 expression predicted better overall survival in those patients with low antioxidant expression (Figure 2F-2I). Taken together, these results demonstrated that SETD7 might be a positive regulator of NRF2 target genes through modulating KEAP1-NRF2 pathway.

### SETD7 regulates the proliferation and survival of breast cancer cells

**Figure 3:**
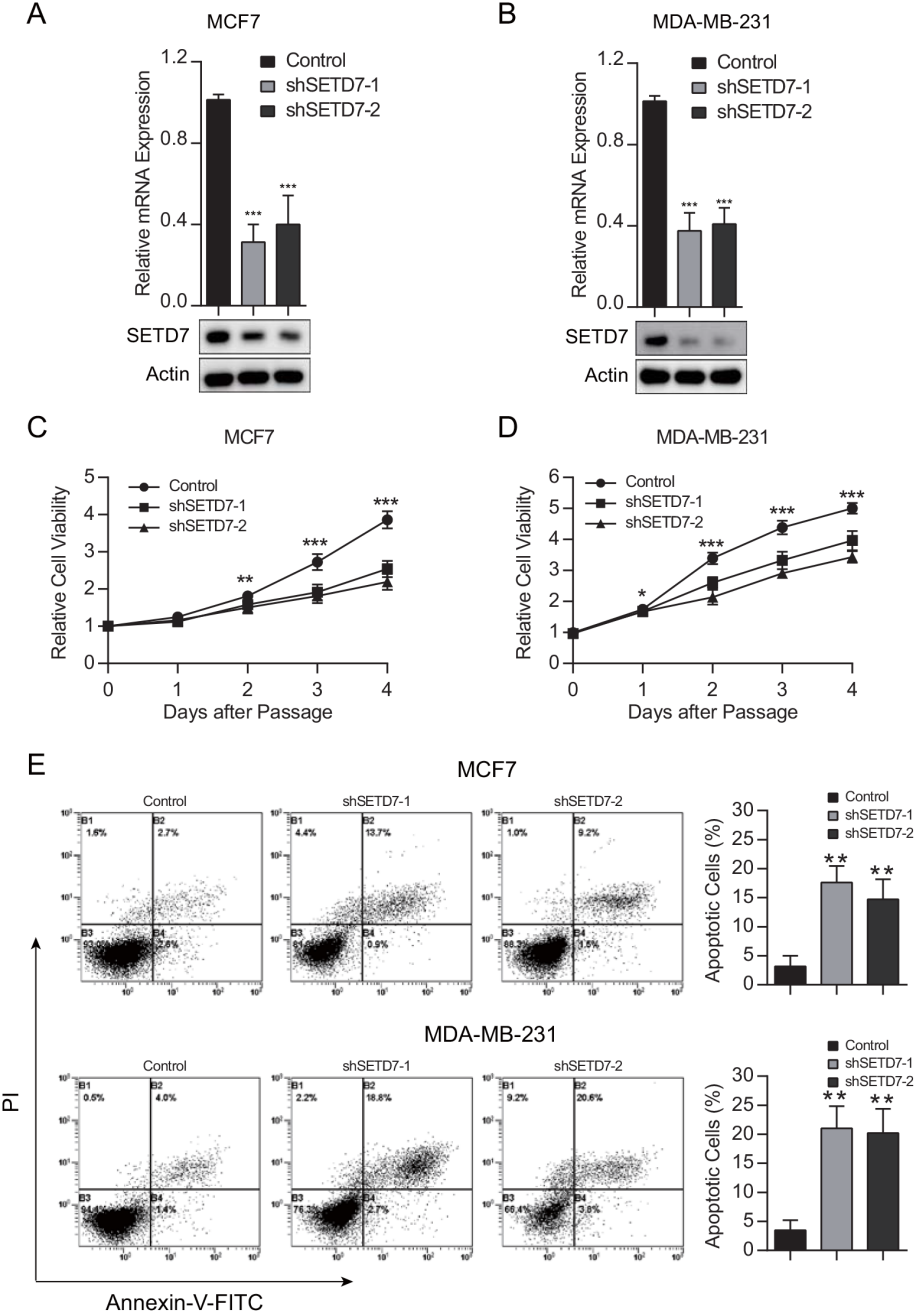
SETD7 promotes cell growth and survival **(A** and **B)** Knockdown efficiency of SETD7, proved by quantitative real-time PCR and western blot analysis. **(C** and **D)** Cell viability was measured in breast cancer cells with depleted SETD7 expression (n=6). **(E** and **F)** Cell were stained with Annexin V–FITC and PI and analyzed by flow cytometry (n=3). Augmented apoptotic effect was observed in the upper right and lower right quadrants when SETD7 was knocked down. ^*^P< 0.05, ^**^P< 0.01, and ^***^P< 0.001. All experiments were repeated twice.

To gain insight into the biological effect of SETD7 on breast cancer cells, we stably knocked down SETD7 expression in two parent breast cancer cell lines MCF7 and MDA-MB-231. The knockdown efficiency was verified by quantitative real-time PCR and western blot analysis (Figure 3A and 3B). To ascertain the contribution of SETD7 to proliferation in breast cancer cells, CCK-8 proliferation assay was performed. Cell viability was significantly attenuated when the expression of SETD7 was knocked down (Figure 3C and 3D). To detect the effect of SETD7 on cancer cell survival, cells were subjected to flow cytometry. Increased apoptotic effect was detected in SETD7-silenced breast cancer cells (Figure 3E). Taken together, these results validated the role of SETD7 promoting cell proliferation and survival.

### SETD7 regulates the redox status of breast cancer cells

**Figure 4:**
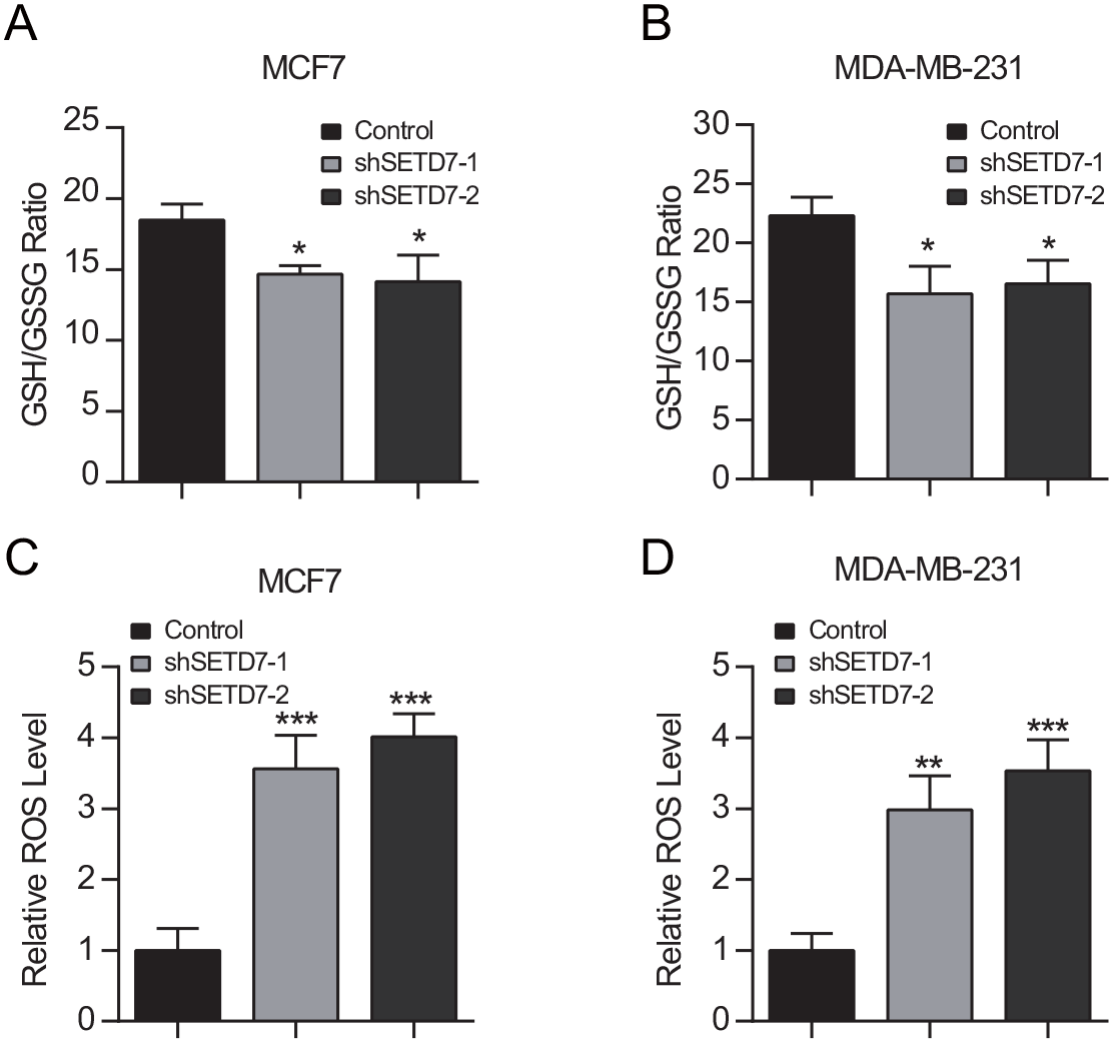
SETD7 maintains intracellular redox status **(A** and **B)** Intracellular GSH and GSSG were determined. SETD7 elevated GSH/GSSG ratio in breast cancer cells (n=3). **(C** and **D)** Attenuated SETD7 expression increased intracellular ROS levels (n=3). ^*^P< 0.05, ^**^P< 0.01, and ^***^P< 0.001. All experiments were repeated twice.

Next, we sought to determine the contribution of SETD7 to redox homeostasis. The GSH/GSSG ratio was significantly lower in SETD7-silenced cancer cells than that of control cells, suggesting that SETD7 maintained the reductive status of cells (Figure 4A and 4B). To directly detect the oxidative stress level of cancer cells, intracellular ROS was examined. In consistent with GSH/GSSG ratio levels, SETD7 knockdown significantly elevated intracellular ROS levels, suggesting that cancer cells with depleted SETD7 expression were subjected to more severe oxidative stress (Figure 4C and 4D). These results demonstrated that SETD7 plays a critical role in redox balance of breast cancer cells.

### SETD7 promotes the transcription of NRF2 target genes

**Figure 5:**
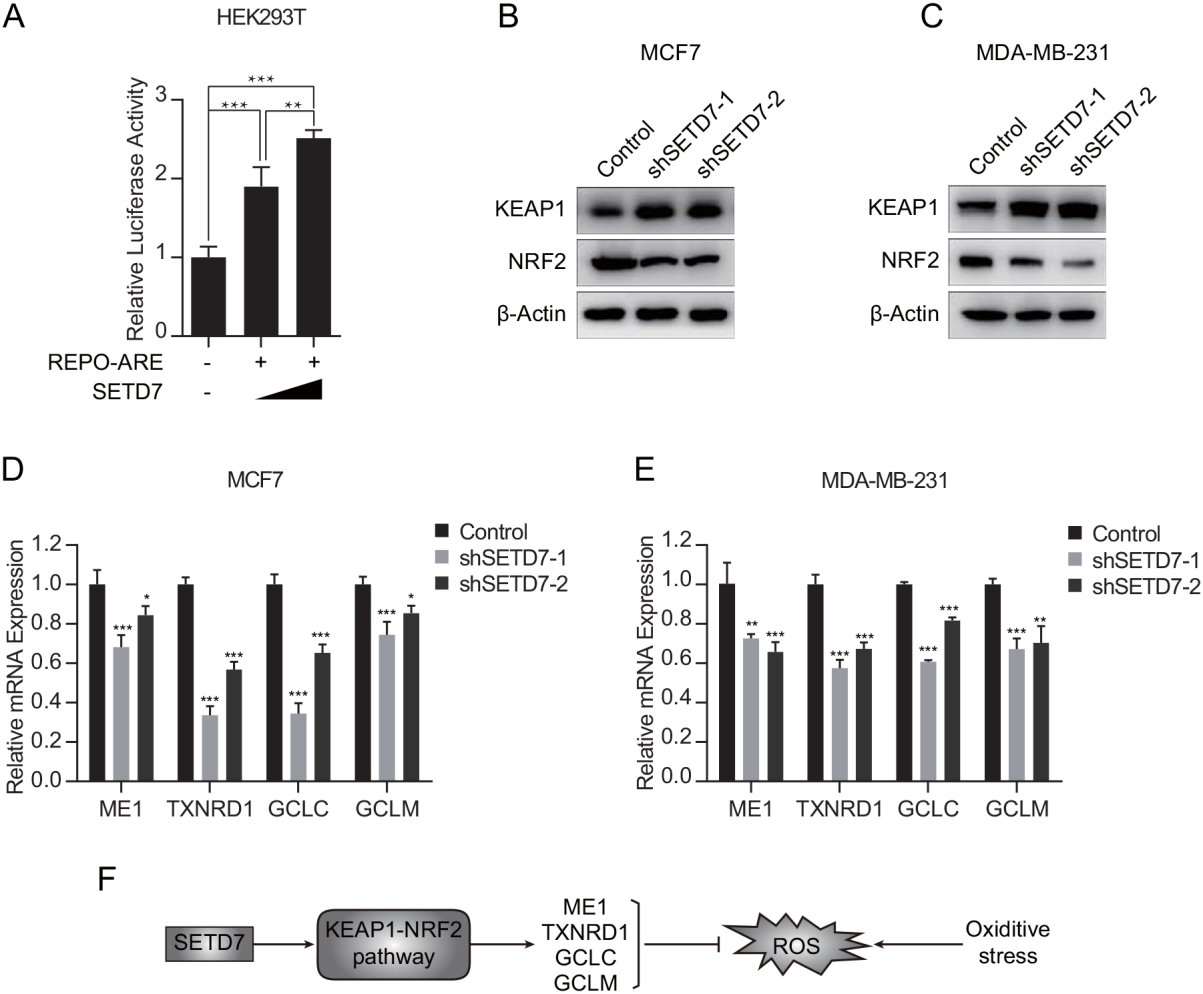
SETD7 promoted the transcription of NRF2 target genes **(A)** Luciferase reporter assay indicated that SETD7 promoted the ARE reporter activity (n=6). **(B** and **C)** Knockdown of SETD7 upregulated KEAP1 expression and downregulated NRF2 expression. **(D** and **E)** Knockdown of SETD7 decreased the expression of antioxidant genes, including ME1, TXNRD1, GCLC and GCLM (n=3). **(F)** Schematic representation of SETD7 regulating antioxidative response. ^*^P< 0.05, ^**^P< 0.01, and ^***^P< 0.001. All experiments were repeated twice.

To further explore the mechanism of SETD7 modulating antioxidant expression, a dual-luciferase reporter assay was employed in HEK293T cells. Cells were cotransfected with the reporter plasmid ARE-luciferase construct and SETD7-expression plasmid. Dual luciferase assay demonstrated that ARE-luciferase activity was modulated by SETD7 in a dose-dependent manner (Figure 5A). Due to the negative correlation between SETD7 and KEAP1 observed from TCGA database, *in vitro* assay was performed to determine the alterations of KEAP1-NRF2 pathway. Attenuation of SETD7 expression increased the expression of KEAP1 and simultaneously inhibited NRF2 expression (Figure 5B and 5C). Moreover, quantitative real-time PCR assay revealed that SETD7 depletion decreased the expression of ARE target genes, including ME1, TXNRD1, GCLC and GCLM (Figure 5D and 5E). These results suggested that SETD7 was an upstream transcriptional regulator of antioxidant proteins, which was dependent on KEAP1- NRF2 pathway.

## DISCUSSION

Breast cancer has been expected to be the most common cancer diagnosed in the United States in 2016, accounting for 29% of all new cancer diagnoses in women [19]. Five-year survival rate of breast cancer is around 85.4%-91.4% according to the race and ethnicity of the patient [20]. Various markers have been reported to predict the prognosis of breast cancer. However, the prognostic value of monomethyltransferase SETD7 has never been explored. Here in this study, we explored the data of breast cancer from TCGA database and we demonstrated the relationship between SETD7 and poor outcome of breast cancer patients.

To explore the mechanism of SETD7 resulting in poor outcome, we analyzed the RNAseq data and clinical data from TCGA database, a tight relevance between SETD7 and KEAP1-NRF2 pathway has been discovered. The KEAP1-NRF2 pathway is of vital importance in modulating intrinsic redox homeostasis [21]. Recently, Hartikainen et al. revealed that both the proteins KEAP1 and NRF2 in the stress response pathway were implicated in breast cancer risk and patient outcome [22, 23]. Targeting the KEAP1-NRF2 pathway was reported to reverse chemotherapy resistance and enhance ROS-related cytotoxicity [24]. In addition, increased SETD7 expression was reported to be involved in metastasis, recurrence and poor prognosis in hepatocellular carcinoma [25]. In our study, we have proved that SETD7 could augment cell growth rate and induce anti-apoptotic effect in breast cancer.

Although several studies have revealed that inhibition of SETD7 improved ROS clearance through modulating the KEAP1-NRF2 pathway, these studies failed to explain the relationship between SETD7 and ROS in tumor cells [26, 27]. Tumor cells, with specific DNA mutations, transformational chromosome structures and aberrant gene expression profile, might be disparate with normal cells. In the context of tumor, however, we found a significantly negative relationship between SETD7 and KEAP1 both in the TCGA database and in experimental discoveries. Depletion of SETD7 significantly elevated the expression of KEAP1 and simultaneously attenuated NRF2 expression, which resulted in the inactivation of this pathway and inability of ROS scavenging. Moreover, SETD7 expression, together with the expression of downstream antioxidants, served as a good predictor of breast cancer prognosis.

Downstream targets of the KEAP1-NRF2 pathway are various antioxidants, whose expressions avail cancer cells to adapt to radiotherapy-induced oxidative stress [28]. Several antioxidant enzymes were examined in this study, including ME1, TXNRD1, GCLC and GCLM. ME1 is a nicotinamide adenine dinucleotide phosphate (NADP)-dependent malic enzyme which produces NADPH that can be used for antioxidation [29]. TXNRD1, a member of the family of pyridine nucleotide oxidoreductases, is also important for cellular redox balance [30]. As for GCLC and GCLM, both of them are biosynthetic enzymes of glutathione (GSH), which is critical for antioxidative reactions and homeostasis maintenance [16]. Particularly, Lien et al. reported that GSH was required for the tumor proliferation and resistance to oxidative stress and chemotherapy in breast cancer [16]. And genetic knockout of *Gclm* (biosynthetic enzyme of GSH in mice) significantly impaired the tumorigenesis in mouse mammary glands [31]. In our work, we demonstrated that SETD7 might be an upstream regulator of this pathway and that SETD7 promoted the transcription of these target antioxidants. Due to the expression of these ROS-detoxifying enzymes, intracellular ROS decrease to lower levels, that is essential for cancer cell proliferation, migration and survival under stressed circumstances [32]. Thus, depicting the role of SETD7 inducing expression of antioxidants will deepen our understanding of breast cancer progression.

In conclusion, we demonstrated that SETD7 was a predictor of breast cancer prognosis and that SETD7 activated the antioxidant pathway and promoted the expression of antioxidant enzymes. Excessive ROS are detrimental to cancer cells, while a well-balanced redox status is required for tumor initiation and resistance to oxidative stress of breast cancer [16]. SETD7 is an epigenetic factor that regulates the posttranslational modifications of histone 3 and a variety of non-histone proteins. In our study, we proposed that SETD7 acts as an activator of antioxidant pathway under stressed conditions in breast cancer (Figure 5F). However, further studies are needed to explore the exact mechanisms of SETD7 in activating the antioxidant response. Deciphering the mechanism of SETD7 in ROS detoxification might help exploit the potential of SETD7 as a therapeutic target for breast cancer.

## MATERIALS AND METHODS

### Cells and reagents

Human embryonic kidney 293T (HEK293T) cell line was obtained from American Type Culture Collection (ATCC). MDA-MB-231 and MCF7 cell lines were purchased from Cell Bank of the Chinese Academy of Sciences (Shanghai, China). HEK293T and MCF7 were cultured in Dulbecco’s modified Eagle’s medium (DMEM) with 10% fetal bovine serum (FBS). MDA-MB-231 was cultured in Leibovitz's L-15 Medium with 10% FBS. To prevent bacteria contamination, Penicillin and Streptomycin were added to the medium at a final concentration of 100 U/mL and 100 mg/mL, respectively. Cells were incubated at 37 °C in a humidified CO_2_ incubator.

### Plasmids

The pLKO.1 TRC cloning vector (Addgene plasmid 10878) was used to generate SETD7 silencing lentiviral vectors. The 21-bp targets against SETD7 were CTTATGAATCAGAAAGGGTTT and GCCCTATAACCACGTATCCAA respectively. The DNA sequences of plasmid constructs were validated by nucleotide sequencing.

### Cell viability and cell apoptosis assay

Cells were seeded onto 96-well plates with each well containing 4500 cells and 100μL culture medium. CCK-8 (Cell Counting Kit-8; Dojindo Laboratories, Kyushu, Japan) was employed to examine the cell viability according to the manufacturer’s instructions.

Apoptosis was detected using AnnexinVfluorescein isothiocyanate (FITC)/propidium iodide (PI) apoptosis detection kit I (BD, San Jose, CA, United States). All the cells, including floating and adherent cells, were collected by centrifugation, washed twice with cooled PBS, and resuspended with 100μL binding buffer per 1×10^5^ cells. Following incubation with 5μL Annexin VFITC and 5μL PI solution in the dark at room temperature for 15 min, 400μL binding buffer was added and shaken slightly. Afterwards, flow cytometry was employed to detect the cell signal using FACS LSR II (BD, USA).

### Dual-luciferase reporter assay

Cells were seeded onto 96-well plates at an initial density of 2×10^4^ cells/well. Totally, 2μg plasmids were transfected into each well. Cells were transfected with 0.5μg of promoter-luciferase plasmid REPO-ARE and 0.5μg/1μg pCMV-SETD7-flag. To normalize transfection efficiency, 60ng Renilla luciferase plasmid was transfected into each well simultaneously. Forty-eight hours after transfection, luciferase activity was determined using the Dual-Luciferase Assay System (Promega). The scheduled experiments were repeated thrice.

### Western blot

Western blotting was performed according to the previously published paper [33]. Briefly, whole-cell protein lysates were extracted and protease inhibitors were added. BCA protein assay was performed to detect the protein concentration. After electrophoresis, proteins were transferred to a PVDF membrane. Then the membrane was blocked using skim milk and incubated with specific antibodies. Antibody against SETD7, KEAP1 and NRF2 was purchased from Proteintech. β-actin was used as a loading control.

### Quantitative real-time PCR (qRT-PCR)

RNA was isolated using Trizol according to manufacturer instructions (Invitrogen, USA). Complementary DNA was synthesized by reverse transcription using a TaKaRa PrimeScript RT reagent kit and then subjected to quantitative real-time PCR using ABI 7900HT Real-Time PCR system (Applied Biosystems, USA). Each reaction was performed in triplicate. Primer sequences used in this experiment are as follows: ME1: 5'-CCTCACTACTGCTGAGGTTATAGC-3' and 5'-CGGTTCAGGATAAACTGTGGCTG-3'; TXNRD1: 5'-GCAATCCAGGCAGGAAGATTGCT-3' and 5'-CTCTTGACGGAATCGTCCATTCC-3'; GCLC: 5'-GTGGTACTGCTCACCAGAGTG-3' and 5'-AGCTCCGTGCTGTTCTGGGCCTT-3'; GCLM: 5'-ATCTTGCCTCCTGCTGTGTGATGC-3' and 5'-CAATGACCGAATACCGCAGTAGCC-3'.

### GSH/GSSG and ROS detection

Cells were collected after washed thrice with phosphate buffer. GSH and GSSG Assay Kit were purchased from Beyotime, China. The determination of GSH and GSSG was performed according to the manufacturer’s instructions. Intracellular ROS was determined using the Reactive Oxygen Species Assay Kit purchased from Beyotime, China according to the manufacturer’s instructions.

### TCGA data acquiring and statistical analysis

Normalized RSEM RNASeqV2 data and clinical data of breast cancer were downloaded from The Cancer Genome Atlas (TCGA) using TCGA Assembler [34]. 1086 patients with RNAseq data and clinical survival data were included in this study (Supplementary Table 1). Correlation analysis was employed to examine the relationships between SETD7 and key molecules of KEAP1-NRF2 pathway and ARE target proteins. Log-rank survival analysis was employed to analyze the effect of SETD7 expression and the cross effect between SETD7 and ARE target genes on breast cancer survival. The distribution differences between SETD7 expression and clinicopathological characteristics examined using chi-square test. The effects of clinicopathological characteristics on survival were firstly examined using Log-rank survival analysis, and univariate COX proportional regression was employed to calculate the hazard ratios (HRs) and 95% confidence intervals (95% CIs). Statistically significant variables in univariate analysis were then entered multivariate COX analysis to build a mathematical model using a stepwise conditional method.

All experimental data were presented as mean ± standard deviation. One-way analysis of variance (ANOVA) was performed to compare these data between each group and Dunnett's test was employed to avoid family-wise error. A *P* value less than 0.05 was considered to be statistically significant in this study. Statistical software SAS 9.4 was used to analyze all the data.

## SUPPLEMENTARY MATERIALS TABLE





## Abbreviations

ARE: antioxidant response element
DNMT1: DNA cytosine methyltransferase 1
E2F1: E2 promoter-binding factor 1
GCL: glutamate-cysteine ligase
HIF1α: hypoxia-inducible factor 1α
KEAP1: Kelch-like ECH-associated protein 1
ME1: malic enzyme 1
NRF2: nuclear factor E2-related factor 2
ROS: reactive oxygen species
SETD7: SET domain containing lysine methyltransferase 7
TCGA: The Cancer Genome Atlas
TXNRD1: thioredoxin reductase 1
