# Supplementary material for: SETD7 is a prognosis predicting factor of breast cancer and regulates redox homeostasis
Source: Oncotarget. 2017 Oct 6;8(55):94080–90. doi: 10.18632/oncotarget.21583 (PMC5706857; doi:10.18632/oncotarget.21583)
Supplement: Supplementary file 1 [file oncotarget-08-94080-s001.pdf]

SETD7 is a prognosis predicting factor of breast cancer and regulates redox homeostasis

SUPPLEMENTARY MATERIALS

Supplementary Table 1: Clinical survival and RNAseq data in TCGA database

See Supplementary File 1
